# An Overview of the Systematic Evidence on the Adjunctive Use of Laser Therapy in Non-surgical Periodontal Treatment

**DOI:** 10.7759/cureus.44268

**Published:** 2023-08-28

**Authors:** Ibrahim A AlZoubi

**Affiliations:** 1 Department of Preventive Dentistry, College of Dentistry, Jouf University, Al Jouf, SAU

**Keywords:** systematic reviews, treatment outcomes, evidence-based study, non-surgical periodontal treatment, adjunctive laser therapy

## Abstract

This overview aimed to recapitulate the evidence related to laser application in non-surgical periodontal treatment along with conventional periodontal treatment for optimum clinical practice based on the available systematic reviews (SRs). An advanced literature search in the English language was conducted in the PubMed, Medical Literature Analysis and Retrieval System Online (MEDLINE), ScienceDirect, and Scopus databases from January 2000 to October 2022. Two independent reviewers screened all the databases and extracted the data in duplicate. The risk of bias in the selected studies was assessed with the Methodological Quality of Systematic Reviews 2 (AMSTAR 2) guideline for SRs. Cohen’s kappa statistics were performed to assess the level of agreement for the assessment of the risk of bias.

A total of 556 studies (PubMed = 115, Scopus = 66, ScienceDirect = 298, and MEDLINE = 77) were identified after the initial search using the keywords from different databases. After removing the duplicates and assessing the full manuscripts, a total of 24 studies were selected based on the inclusion criteria for the current overview. A total of three, four, 12, and five systematic reviews were classified as high, moderate, low, and critically low-quality SRs as per the AMSTAR 2 quality assessment tool. Cohen’s Kappa statistics showed perfect (𝛋 =1.000) agreement between the two reviewers. Adjunctive laser therapy along with conventional non-surgical periodontal treatment might be effective in short-term treatment outcomes; however, evidence of long-term effects is still lacking.

## Introduction and background

Periodontitis is an infectious condition caused by inflammatory pathogens [1]. This phenomenon is generally treated with either surgical or non-surgical modalities. Scaling and root planning (SRP) is one of the most common techniques to conduct non-surgical treatment modalities for periodontitis [2,3]. The outcome of SRP is also influenced by patient-related factors such as systemic disease, smoking, and stress [4-6]. Other than patient-related factors, sometimes ultrasonic or manual instruments fail to access the furcation area, deep pockets area, and groove, resulting in incomplete removal of detriments [7]. Moreover, unfavorable wounds in periodontal tissue can also be observed after the SRP treatment [8]. Therefore, alternative treatments for periodontitis have been investigated in conjunction with SRP.

A few alternatives, such as local application of antiseptic, antibiotic, and laser (light amplification by stimulated emission of radiation) therapy, were proposed as a supplement or substitute for non-surgical periodontal treatment [9-11].

Laser therapy is a non-invasive procedure that is rarely associated with any adverse effects in periodontal treatment. Different types of lasers with diverse wavelengths were commonly studied in the management of periodontitis with SRP [12-14]. Diode laser (DL), erbium, chromium-doped: yttrium, scandium, gallium, garnet (Er, Cr: YSGG) laser, neodymium-doped yttrium aluminum garnet (Nd: YAG) laser, erbium-doped: yttrium, aluminum, and garnet (Er: YAG) laser, and carbon dioxide (CO2) laser are the most commonly used as adjunctive non-surgical periodontal treatment [15]. Various independent studies investigated the efficacy of laser therapy in periodontal treatment; however, the outcome was not consistent. Therefore, systematic reviews (SRs) were conducted to distinguish the pooled efficacy of laser therapy in periodontal treatment [16-20].

Though there is accessibility to systematic reviews, effective clinical practices of laser therapy have failed due to a lack of knowledge. In addition, there is a lack of consensus among professionals [8, 21, 22]. Therefore, The Cochrane Collaboration proposed a new study design, the overview of systematic reviews, where the outcome of various systematic reviews is assembled in a single study [23]. An overview of systematic reviews provides easy access to the combined information and forms a hierarchy of evidence that aids in decision-making [24, 25]. Hence, the current study is based on systematic reviews, which are considered the highest level of scientific evidence. This overview of systematic reviews aimed to recapitulate the evidence related to laser application in non-surgical periodontal treatment regarding the optimum clinical practice.

## Review

An advanced literature search in the English language was conducted in the PubMed, Medical Literature Analysis and Retrieval System Online (MEDLINE), ScienceDirect, and Scopus databases from January 2000 to October 2022. Two independent reviewers screened all the databases in duplicate using the following keywords: (‘periodontitis’ OR ‘periodontal disease’ OR ‘non-surgical periodontal treatment’ OR ‘periodontal treatment’) AND (‘laser’ OR ‘laser therapy’ OR ‘photodynamic therapy’) AND (‘systematic review’ OR 'meta-analysis’). Manual hand searching was also performed from the reference list of the selected studies. All the selected titles and abstracts were managed with the Endnote software (version X9). The overview was performed according to the Preferred Reporting Items for Systematic Reviews and Meta-Analyses (PRISMA) guidelines by Moher et al. [26].

The following inclusion criteria were followed for selecting the studies in this overview: SRs with or without meta-analysis, non-surgical periodontal treatment such as SRP, and application of laser therapy as an adjunct to the periodontal treatment. On the other hand, studies other than SRs, surgical treatment of periodontitis, and adjunctive treatment with antimicrobial photodynamic therapy were excluded from this overview.

**Figure 1:**
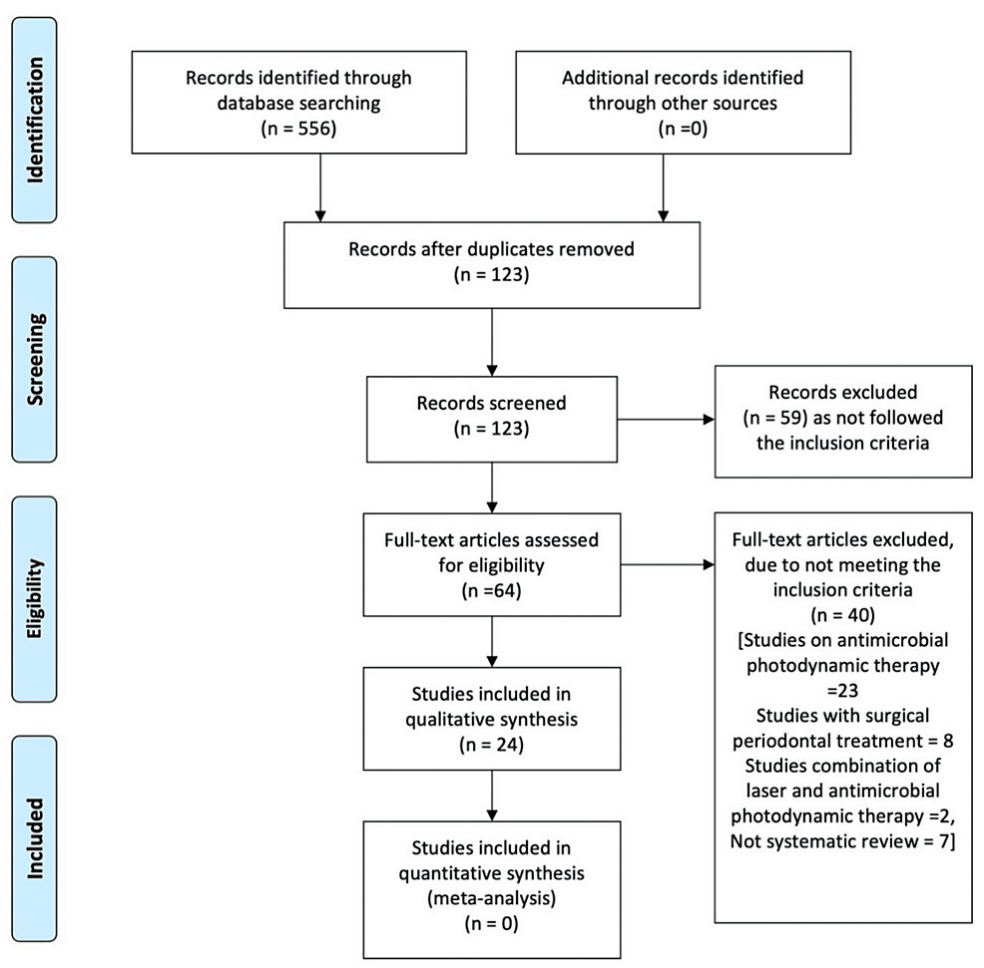
A flowchart of the study selection as per the PRISMA guidelines PRISMA: Preferred Reporting Items for Systematic Reviews and Meta-Analyses

The titles and abstracts were screened primarily by two independent reviewers. The full manuscripts were selected if the studies met the inclusion criteria. After removing duplicates, full texts were evaluated by two reviewers who independently listed all the studies to be included in this review (Figure 1).

Both reviewers resolved any disagreements with a discussion regarding finalizing the studies. Both reviewers checked the selected lists of studies. If the final lists by both reviewers did not match, they discussed the reason for the elimination of the study and decided whether to keep it in the final list of selected studies by consensus. The risk of bias in the selected studies was assessed with the Methodological Quality of Systematic Reviews 2 (AMSTAR 2) guideline for SR (Table 1).

**Table 1:** AMSTAR 2 assessment *Critical domain item High (no, or one non-critical weakness: the systematic review provides an accurate and comprehensive summary of the results); moderate (more than one non-critical weakness but no critical flaws: the systematic review provides an accurate summary of the results); low (one critical flaw, with or without non-critical weaknesses: the systematic review may not provide an accurate and comprehensive summary of the results); critically low (more than one critical flaw, with or without non-critical weaknesses: the review should not be relied on to provide an accurate and comprehensive summary of the results). PICO:  patient/population, intervention, comparison, and outcomes; AMSTAR 2:  Methodological Quality of Systematic Reviews 2

Question	AMSTAR 2 item
Q1	Did the research questions and inclusion criteria for the review include the components of PICO?
Q2*	Did the report of the review contain an explicit statement that the review methods were established prior to the conduct of the review, and did the report justify any significant deviations from the protocol?
Q3	Did the review authors explain their selection of the study designs for inclusion in the review?
Q4*	Did the review authors use a comprehensive literature search strategy?
Q5	Did the review authors perform study selection in duplicate?
Q6	Did the review authors perform data extraction in duplicate?
Q7*	Did the review authors provide a list of excluded studies and justify the exclusions?
Q8	Did the review authors describe the included studies in adequate detail?
Q9*	Did the review authors use a satisfactory technique for assessing the risk of bias in individual studies that were included in the review?
Q10	Did the review authors report on the sources of funding for the studies included in the review?
Q11*	If meta-analysis was performed, did the review authors use appropriate methods for the statistical combination of results?
Q12	If a meta-analysis was performed, did the review authors assess the potential impact of the risk of bias in individual studies on the results of the meta-analysis or other evidence synthesis?
Q13*	Did the review authors account for the risk of bias in individual studies when interpreting or discussing the results of the review?
Q14	Did the review authors provide a satisfactory explanation for and discussion of any heterogeneity observed in the results of the review?
Q15*	If they performed quantitative synthesis, did the review authors carry out an adequate investigation of publication bias (small study bias) and discuss its likely impact on the results of the review?
Q16	Did the review authors report any potential sources of conflict of interest, including any funding they received for conducting the review?

A total of 16 items were used to evaluate the methodological quality of the SR. Each item was scored as ‘yes’, ‘partially yes’, ‘no’, and ‘no meta-analyses’. Seven out of 16 items were considered critical domains (item two, item four, item seven, item nine, item 11, item 13, and item 15) (Table 1). Each study scored as high (no or one non-critical weakness), moderate (more than one non-critical weakness but no critical flaws), low (one critical flaw with or without non-critical weakness), and critically low (more than one critical flaw with or without non-critical weakness). A level of agreement between the two reviewers was achieved. The clinical heterogeneity of the selected SRs was assessed. Based on the similar types of lasers and the follow-up duration of the periodontal measurements, the clinical effects of adjunctive laser therapy were evaluated.

Cohen’s kappa statistics were performed to assess the level of agreement for the assessment of the risk of bias. Kappa (𝜅) scores were categorized as 0.81-1 (near to perfect), 0.61-0.80 (substantial), 0.41-0.60 (moderate), 0.21-0.40 (fair), and 0-0.20 (poor) [27]. Statistical analyses were performed using IBM SPSS statistics software (version 27) for macOS (IBM Co., Armonk, NY, USA).

A total of 556 studies (PubMed = 115, Scopus = 66, ScienceDirect = 298, and MEDLINE = 77) were identified after the initial search using the keywords from different databases. After removing the duplicates and assessing the titles and abstracts, a total of 64 studies were selected for full-text assessment. Two independent reviewers screened and assessed all the full texts and finally selected 24 studies based on the inclusion criteria to be included in the current overview (Table 2).

**Table 2:** Characteristics of included studies N: total number of studies; MA: meta-analysis; RCT: randomized controlled trial; NS: not specified; PD: periodontal depth; CAL: clinical attachment level; BOP: bleeding on probing; PI: plaque index; GI: gingival index; REC: recession; Er: YAG: erbium-doped: yttrium, aluminum, and garnet; DL: diode laser; Nd: YAG: neodymium-doped yttrium aluminum garnet; CCT: clinical controlled trial; VAS: visual analog scale; pros: prospective study; Er,Cr: YSGG: erbium, chromium-doped: yttrium, scandium, gallium, garnet; LLLT: low-level laser therapy

Author (year)	Study design	N	Types of lasers	Measurements	MA	Follow-up	Conclusion
Cheng Y [8] (2015)	RCTs, quasi-RCTs	11	NS	PD, CAL, BOP	Yes	Three to six months	Adjunctive laser therapy might be effective in reducing PD and BOP in a relatively shorter term (three months) without detecting any adverse effects.
Coluzzi D [16] (2020)	RCTs	20	NS	NS	No	Six months	Lasers have an adjunctive role in initial non-surgical periodontal therapy.
Jiang Y [17] (2020)	RCTs	16	NS	PD	Yes	Six months	Laser-assisted non-surgical treatment improved clinical outcomes compared to SRP alone in the management of non-smoking chronic periodontitis patients.
Lin Z [18] (2021)	RCTs, CCTs	8	NS	PPD, CAL, BOP, PI	Yes	≤ Six months	In untreated periodontitis patients, laser monotherapy does not yield superior clinical benefits compared with non-surgical mechanical instrumentation alone.
Pawelczyk- Madalińska [19] (2021)	RCTs	15	DL	PD, GI, PI, BOP, PPD, CAL, MGI	No	NS	The efficacy of adjunctive diode laser treatment ranging between 808 and 980nm for SRP remains debatable.
Salvi G [20] (2020)	RCTs, CCTs	17	NS	PD	Yes	Six months	Available evidence on adjunctive therapy with lasers is limited by (i) the low number of controlled studies and (ii) the heterogeneity of study designs.
Sgolastra F [21] (2012)	RCTs	5	Er: YAG	CAL, BOP, PD, PI, GI	Yes	< Six months	No significant difference was observed in the clinical parameter.
Trombelli L [28] (2020)	RCTs	3	Er: YAG	CAL	Yes	One year	Weak evidence indicates that in treated periodontitis patients enrolled in a three- to four-month SPT based on PMPR, Er:Yag laser (as an alternative) does not produce a greater clinical effect on periodontal conditions compared to PMPR.
Schwarz F [29] (2008)	RCTs, CCTs	12	NS	Clinical data	No	NS	The Er:YAG laser seems to possess characteristics most suitable for the non-surgical treatment of chronic periodontitis
Sgolastra F [30] (2013)	RCTs	5	DL	CAL, BOP, PD, PI, GI	Yes	< Six months	The use of DL adjunct with conventional non-surgical treatment did not provide any clinical benefit.
Sgolastra F [31] (2014)	RCTs	3	Nd: YAG	CAL, BOP, PI	Yes	NS	The evidence is insufficient to support the effectiveness of adjunctive Nd: YAG and SRP.
Chambrone L [32] (2018)	RCTs	28	NS	PD, CAL, BOP, REC	Yes	≥ Three months	Clinical improvements in PD and CAL are attributable to infrared laser procedures when used alone or as an adjunct to other periodontal therapy.
Zhao Y [33] (2014)	RCTs	12	Er: YAG	Clinical outcome	Yes	Three months	The clinical efficacy of Er: YAG laser was similar to SRP three months postoperatively. The clinical benefits of Er: YAG laser as an adjuvant to SRP were still lacking.
Yu S [34] (2022)	RCTs	30	NS	PD, CAL, BOP, PI, GI	Yes	Four to six weeks, Three, six months	Adjunctive DL had additional clinical benefits in the treatment of periodontitis.
Slot DE [35] (2014)	RCTs	9	DL	PD, CAL, GI	Yes	NS	The collective evidence regarding adjunctive use of the DL with SRP indicates that the combined treatment provides an effect comparable to that of SRP alone.
Ren C [36] (2017)	RCTs	7	LLLT	PPD, CAL, GI, PI	Yes	NS	The LLLT showed only short-term additional benefits after conventional SRP. The long-term effect remains unclear.
Li M [37] (2021)	RCTs	16	Er,Cr: YSGG	PD, CAL, VAS	Yes	NS	The laser application showed clinical effectiveness in short-time follow-up, but no significant difference in the six-month follow-up.
Jia L [38] (2020)	RCTs	25	NS	CAL	Yes	Three, six months	Laser-assisted periodontal treatment could be superior to SRP alone and could serve as a good adjunctive treatment tool.
Slot D [39] (2009)	RCTs	8	Nd: YAG	PI, BOP, GI, PD	No	NS	No evidence to support the superiority of the Nd: YAG laser over traditional modalities of periodontal therapy.
Roncati M [40] (2014)	RCTs, CCTs, Pros.	6	Nd: YAG/ DL	PD, BOP, CAL	Yes	≥ Six months	Use of the Nd: YAG or diode laser in conjunction with nonsurgical periodontal therapy, as it seems to provide an adjunctive benefit in terms of PPD and BOP compared with mechanical debridement
Qadri T [41] (2015)	Clinical studies	27	DL	PI, BOP, CAL, PD	No	NS	In CP patients with probing depths £5 mm, diode lasers, SRP plus diode laser (800–980 nm) is more effective in the treatment of CP than when SRP is used alone
Ma L [42] (2018)	RCTs	12	Er: YAG	PD, CAL, VAS	Yes	Three, six, 12 months	This systematic analysis demonstrated that ERLs+SRP provides additional short-term effectiveness and that patients experience less pain compared to SRP. There were no significant differences at the medium-term and long-term follow-ups. Long-term well-designed RCTs are required.
Karlsson M [43] (2008)	RCTs	4	NS	PD, CAL, BOP	No	NS	No consistent evidence supports the efficacy of laser treatment as an adjunct to non-surgical periodontal treatment in adults with chronic periodontitis
Estrin N [44] (2022)	RCTs	4	Nd: YAG, Er: YAG	PD, CAL, REC	Yes	NS	Combination of Nd:YAG and Er:YAG could clinically improve than a single methods

The rest of the studies were excluded, and the reasons for exclusion are mentioned in Figure 1.

Risk of bias assessment

The AMSTAR 2 tool was used to evaluate the risk of bias for all selected SRs, and the assessment is presented in Table 3.

**Table 3:** Quality analysis using the AMSTAR 2 tool Q1-Q16: explained in table 1; Y: yes; N; no; NMA: no meta-analysis; PY: partially yes; Cr: critically

Studies	Q1	Q2	Q3	Q4	Q5	Q6	Q7	Q8	Q9	Q10	Q11	Q12	Q13	Q14	Q15	Q16	Quality
Cheng Y [8] (2015)	Y	Y	Y	Y	Y	Y	PY	Y	Y	N	Y	Y	Y	Y	Y	Y	High
Coluzzi D [16] (2020)	N	Y	Y	Y	N	Y	N	Y	Y	N	NMA	NMA	Y	N	NMA	N	Low
Jiang Y [17] (2020)	N	Y	Y	Y	N	Y	N	Y	Y	N	Y	N	Y	Y	Y	Y	Low
Lin Z [18] (2021)	Y	Y	Y	Y	N	Y	Y	Y	Y	Y	Y	Y	Y	Y	N	Y	Low
Pawelczyk- Madalińska [19] (2021)	Y	Y	Y	Y	Y	Y	Y	Y	Y	Y	NMA	NMA	Y	Y	NMA	N	High
Salvi G [20] (2020)	Y	Y	Y	Y	Y	Y	Y	Y	Y	Y	Y	Y	Y	Y	N	N	Low
Sgolastra F [21] (2012)	N	Y	Y	Y	Y	Y	Y	Y	Y	N	Y	Y	Y	Y	Y	N	Mod
Trombelli L [28] (2020)	Y	Y	Y	Y	Y	Y	Y	Y	Y	Y	Y	Y	Y	Y	Y	N	High
Schwarz F [29] (2008)	N	Y	Y	Y	Y	Y	Y	Y	Y	N	NMA	NMA	Y	Y	NMA	N	Mod
Sgolastra F [30] (2013)	N	Y	Y	Y	Y	Y	N	Y	Y	N	Y	Y	Y	N	Y	N	Low
Sgolastra F [31] (2014)	N	Y	Y	Y	Y	Y	Y	Y	Y	N	Y	Y	Y	Y	Y	N	Mod
Chambrone L [32] (2018)	N	Y	Y	Y	N	Y	Y	Y	Y	Y	Y	Y	Y	Y	Y	Y	Mod
Zhao Y [33] (2014)	Y	Y	Y	Y	Y	Y	N	Y	Y	N	Y	Y	Y	Y	Y	N	Low
Yu S [34] (2022)	Y	Y	Y	Y	Y	Y	Y	Y	Y	Y	Y	Y	Y	Y	N	N	Low
Slot DE [35] (2014)	Y	Y	Y	Y	Y	Y	Y	Y	Y	Y	Y	Y	Y	Y	N	N	Low
Ren C [36] (2017)	N	Y	Y	Y	Y	Y	N	Y	Y	N	Y	Y	Y	N	N	Y	Low
Li M [37] (2021)	Y	Y	Y	Y	Y	Y	N	Y	Y	N	Y	N	Y	Y	Y	Y	Low
Jia L [38] (2020)	Y	Y	Y	Y	N	Y	N	Y	Y	N	Y	Y	Y	N	Y	Y	Low
Slot D [39] (2009)	N	Y	Y	Y	Y	Y	N	Y	Y	Y	NMA	NMA	Y	Y	NMA	N	Low
Roncati M [40] (2014)	N	Y	Y	Y	Y	Y	N	Y	N	N	Y	N	N	N	N	N	Cr. Low
Qadri T [41] (2015)	Y	Y	Y	Y	Y	Y	N	Y	N	N	NMA	NMA	N	N	NMA	N	Cr. Low
Ma L [42] (2018)	Y	Y	Y	Y	N	Y	N	Y	Y	N	Y	Y	N	Y	Y	N	Cr. Low
Karlsson M [43] (2008)	N	N	Y	Y	N	Y	N	Y	N	N	NMA	NMA	Y	N	NMA	Y	Cr. Low
Estrin N [44] (2022)	Y	Y	Y	Y	Y	Y	N	Y	Y	N	Y	N	Y	N	N	N	Cr. Low

Three high-quality studies [8, 19, 28] and four moderate-quality studies [29-32] were identified out of a total of 24 studies. Moreover, 12 studies [33, 34, 35, 30, 20, 36, 18, 37, 38, 17, 16, 39] were classified as low quality, and five studies [40- 44] were classified as critically low-quality studies. Both reviewers used the AMSTAR 2 tool for the selected studies and assessed the quality of the studies.

Synthesis of results

The characteristics of the included SR were described according to the author and year of publication, study design, number of studies included, type of laser, measurements, meta-analysis, follow-up, and conclusion, which are presented in Table 2.

A few primary studies overlapped with the included SR. Hence, no meta-analysis was performed in this overview due to the duplication of information that would provide a biased overview [45]. The clinical effects of adjunctive laser therapy along with SRP are presented in Table 4.

**Table 4:** Effects of adjunctive laser therapy for periodontitis from the evidence Er: YAG; erbium-doped: yttrium, aluminum, and garnet; CAL: clinical attachment level; PD: pocket depth; Nd: YAG; neodymium-doped: yttrium aluminum garnet; DL: diode laser; P: P-value *: significant differences (<0.05)

Author	Results on effectiveness	Studies included	P value
Er: YAG (three months)			
Zhao Y [33] (2014)	CAL: 0.13, (CI: -0.49, 0.75)	CAL: 4	P=0.99
	PD: 0.11(CI: -0.34, 0.56)	PD: 4	P=0.88
Ma L [42] (2018)	CAL: 0.35, (CI: 0.41, 0.39)	CAL: 7	P=<0.001*
	PD: 0.32, (CI: 0.14, 0.51)	PD: 7	P=<0.001*
Er: YAG (six months)			
Sgolastra F [21] (2012)	CAL: 0.01, (CI: -0.72, 0.73)	CAL: 4	P=0.99
	PD: -0.03, (CI: -0.45, 0.38)	PD: 4	P=0.88
Ma L [42] (2018)	CAL: -0.01, (CI: -0.21, 0.19)	CAL: 4	P=0.92
	PD: 0.12, (CI: -0.07, 0.31)	PD: 4	P=0.21
Er: YAG (12 months)			
Sgolastra F [21] (2012)	CAL: 0.09, (CI: -1.51,1.68)	CAL: 2	P=0.92
	PD: -0.09, (CI: -1.10, 0.92)	PD: 2	P=0.86
Ma L [42] (2018)	CAL: 0.09, (CI: -0.17,0.36)	CAL: 2	P=0.49
	PD: 0.14, (CI: -0.10, 0.37)	PD: 2	P=0.25
Nd: YAG (six months)			
Chambrone L [32] (2018)	CAL: 1.31, (CI: 0.47,2.15)	CAL: 1	P=0.002*
	PD: 0.45, (CI: -0.83, 1.73)	PD: 2	P=0.49
Roncati M [40] (2014)	CAL: 0.25, (CI: -0.17, 0.68)	CAL: 4	P=0.25
	PD: 0.28, (CI: -2.30, 0.02)	PD: 6	P=0.02*
DL (six months)			
Sgolastra F [30] (2013)	PD: 0.10, (CI: -0.11, 0.31)	PD: 5	P= 0.35
Chambrone L [32] (2018)	PD: 0.63, (CI: 0.27, 0.98)	PD: 2	P=0.0005*
Yu S [34] (2022)	PD: -0.22, (CI: -0.61, 0.16)	PD: 7	P=0.26

None of the pooled effects of previous SR [21,42] exhibited significant differences between the two groups (laser with SRP and SRP only) with Er: YAG laser in six months and 12 months in periodontal pocket depth (PPD) and clinical attachment level (CAL). One meta-analysis showed a significant difference in favor of adjunctive Yr: YAG laser at the three-month follow-ups in PPD and CAL measurements [42]. One out of three meta-analyses found significant effects of diode lasers at the six-month follow-up in PPD [32]. Meta-analysis on Nd:YAG laser presented mixed outcomes. Roncati M et al. (2014) [40] found a significant improvement in adjunctive laser therapy in PPD, whereas CAL showed no significant difference. On the other hand, Chambrone et al. (2018b) [46] showed the opposite pooled effect.

Discussion

Adjunctive use of laser therapy to SRP in periodontitis did not reveal robust evidence from the previous SRs. Though the use of lasers did not report any side effects, it is important to highlight that most of the studies conducted the comparison in short-term follow-up (≤ six months), which is not enough time to conclude about the effectiveness of the laser. As per the results of the SRs, the laser might be effective as an adjunct with SRP for a short period, yet its effectiveness for a longer period is still questionable.

It needs to be mentioned that this overview was based on the published SRs. Therefore, the data exhibited in the tables were gathered from the included SR and meta-analyses. The current overview did not pay any attention to extracting data from the primary studies.

All the SRs included in this overview were selected for randomized clinical trials (RCTs) as the study design. However, two studies by Salvi et al. (2020) [20] and Roncati and Gariffo (2014) [40] included prospective controlled clinical trials (CCTs) along with RCTs. None of the SRs included retrospective studies except for Roncati M et al. (2014) [40]. Oftentimes, a retrospective study design provides weak evidence for a systematic review. Moreover, retrospective study design instigates low scores in quality analysis in SRs due to the blinding factor, which is an important assessment parameter in any quality analysis (Alam et al. (2022) [47] and Raposo et al. (2018) [48]). Therefore, RCTs stipulate a more constructive quality of report in an SR [49].

The number of primary studies included in an SR plays a major influence on the outcome of the SR. Out of the 24 studies included, 10 assessed fewer than 10 primary studies. For instance, Sgolastra F et al. (2014) [31] and Trombelli L et al. (2022) [28] included just three studies each, while Karlsson M et al. (2018) [43] and Estrin N et al. (2022) [44] incorporated four studies each. On the other hand, Sgolastra F et al. (2012) [21] and Sgolastra F et al. (2013) [30] included two primary studies, both by Sgolastra et al. Most of the SRs, with a small number of primary studies, concluded that adjunctive laser therapy has no superior advantages compared to conventional SRP treatment. These were by Trombelli et al. (2020) [28], Slot et al. (2009b) [39], Sgolastra et al. (2014) [31], Sgolastra et al. (2012) [21], Sgolastra et al. (2013) [30], and Karlsson et al. (2008) [43].

In terms of the types of lasers used, the majority of the studies did not specify the laser; nevertheless, a few studies focused on specific laser applications only. A total of four studies focused on only the Er:YAG laser, and they were by Ma et al. (2018) [42], Sgolastra et al. (2012) [21], Trombelli et al. (2020) [28], Zhao et al. (2014) [33], along with SRP, and all the studies showed a negative association with periodontal outcome. Moreover, only Nd:YAG laser application was evaluated in two studies by Slot et al. (2014) [35] and Sgolastra et al. (2014) [31], which also did not find any significant influence on periodontal perimeters compared to conventional SRP treatment protocols. However, Estrin N et al. (2022) [44] conducted a network meta-analysis in a combination of Er:YAG and Nd:YAG laser therapy along with SRP and concluded an improvement in periodontitis [44]. In addition, diode lasers with specific wavelengths were assessed by Qadri T et al. (2015) [41] and Slot DE et al. (2014) [35], where adjunctive DL therapy exhibited improvement in periodontitis treatment compared to SRP by Qadri et al. (2015) [41] and Slot et al. (2014) [35]. The SR that was conducted by including primary studies regardless of the types of lasers concluded a positive outcome of adjunctive laser therapy by Cheng et al. (2016) [8], Coluzzi et al. (2020) [16], Jia et al. (2022) [38], Jiang et al. (2022) [37], and Chambrone et al. (2018a) [32]. The primary studies usually use one type of laser in their studies, irrespective of the study design. Therefore, it would be better to conduct SR with a specific laser with a specific wavelength, which could provide a constructive outcome for a specific type of laser to be used clinically.

Most of the studies concluded that adjunctive laser therapy with conventional SRP is effective compared to the only SRP treatment, except for a few studies that did not identify any positive effects of additional laser therapy over conventional SRP treatment [43, 2, 21, 30, 39, 28]. Moreover, few studies claimed that laser therapy showed clinical effectiveness only in short-term follow-up; however, there was no significant benefit in long-term follow-up [8, 37, 42, 36]. Moreover, among the 24 selected SRs, six studies by Coluzzi et al. (2020) [16], Karlsson et al. (2008) [43], Pawelczyk-Madalińska et al. (2021) [19], Schwarz et al. (2008) [29], Slot et al. (2009a) [39], and Qadri et al. (2015) [41] did not perform a meta-analysis, whereas other studies included meta-analysis regardless of the number of included studies. The number of meta-analyses that assessed the periodontal variable was three, and those were by Trombelli et al. (2020) [28], Sgolastra et al. (2014) [31], and Yu et al. (2022) [34]. This overview extracted a few homogenous meta-analyses in Table 3 on PPD and CAL. The use of laser compared to SRP increased PPD reduction by -0.22 mm by Yu et al. (2022) [34] to 0.63 mm by Chambrone et al. (2018) [32] on average. In addition, the change in CAL varied from -0.01 mm in a study conducted by Ma et al. (2018) [42] to 1.31 mm by Chambrone et al. (2018) [32].

A total of three, four, 12, and five SRs were classified as high, moderate, low, and critically low in the quality analysis by the AMSTAR 2 tool. The AMSTAR tool was first published in 2007 in order to assess the quality of SRs [50]. In 2017, the original AMSTAR tool was revised and modified to AMSTAR 2 for a better reflection of the risk of bias and better alignment of the selected format by Shea et al. (2017) [51]. Using the AMSTAR 2 tool, researchers and clinicians differentiate the high-quality SR from the reviews that were inadequately conducted. Therefore, before conducting an SR, researchers should consider the AMSTAR 2 tool for a better outcome. Moreover, the reviews published after 2017 should have scored higher in the quality assessment, and two SRs, Pawelczyk-Madalińska et al. (2021) [19] and Trombelli et al. (2020) [28], were among the three high-quality SRs in the current overview published after 2017.

Among all the studies that were classified as moderate, none violated any critical assessment (Table 1); however, the most common items that were ignored were items 1 and 10. The number 1 item is based on the participants, intervention, comparison, and outcome (PICO) format for conducting an SR. All the SRs, straightforwardly or subsidiarily, use this format; however, it is imperative to mention it clearly in the SR. On the other hand, item 10 is about the funding. Most of the readers usually ignore the funding part mentioned in the manuscript; nevertheless, it is an important topic, specifically if the study design is an RCT. Therefore, the AMSTAR 2 tool requires this item to be addressed in the SR, though we did not put this item under the critical domain. Most of the studies that were conducted after 2017 usually mentioned the funding item in SR [46, 18-20, 28,34].

According to the AMSTAR 2 tool, only one violation of the critical domain classified any SR as a low-quality study. The majority of the selected SRs in this overview are classified as low-quality SRs. Item 7 and item 15 were the most common critical domains that were not addressed in the respective studies. Item 7 represents the list of exclusion studies, and item 15 signifies publication bias. Previously, excluded studies were just presented with a number; however, the AMSTAR 2 tool recently demonstrated that it could list down the excluded studies with the reasons. Most of the recent studies mentioned the excluded list and explanation either inside the main manuscript or as a supplementary document in the journal. On the other hand, another important factor is publication bias, which lists many SRs as low-quality SR. Analyzing a publication is an important part of a meta-analysis. Many meta-analyses in this current overview were conducted with small numbers of primary studies. Statistical publication bias generally cannot be conducted when the number of included studies is less than 10 [47]. Therefore, many of the included studies did not perform publication bias, which led to low-quality studies. Some of the studies fulfilled all the criteria for the AMSTAR 2 tool except item 15 and dropped into low-quality studies [28, 34, 35].

A total of five SRs-Roncati and Gariffo (2014) [40], Qadri et al. (2015) [41], Ma et al. (2018) [42], Karlsson et al. (2008) [43], and Estrin et al. (2022) [44]-were listed as critically low quality under the AMSTAR 2 tool due to violating more than one critical domain. Items seven, nine, and 10 are the most violated critical domains by these studies. However, all these studies were published before the AMSTAR 2 tool was proposed, except one study by Estrin et al. (2022) [44]; therefore, this overview could not exclude these studies based on the critically low-quality study.

The limitations inherent to this SR include the classification of the detected SR into distinct quality levels ranging from high to severely low based on the AMSTAR 2 assessment tool, which serves as an example of the inherent heterogeneity in methodological quality that systematic reviews as the primary data source involve. The robustness of inferences made from the combined evidence may be impacted by this variability in SR quality. Additionally, even if the AMSTAR 2 guidelines were used to assess the risk of bias in a few particular studies, its accuracy in identifying potential sources of bias might remain subject to concern.

## Conclusions

Based on this overview, though adjunctive laser therapy showed a superior effect in periodontitis patients in short-term follow-up, the long-term effect is not satisfactory. Therefore, long-term studies for laser application along with conventional periodontal treatment are required before clinically treating periodontitis patients with laser.
